# Cancer incidence in the Falkland Islands

**DOI:** 10.1054/bjoc.2001.2099

**Published:** 2001-09-01

**Authors:** A J Swerdlow, B Elsby, Z Qiao

**Affiliations:** Section of Epidemiology, Institute of Cancer Research, D. Block, Cotswold Rd, Sutton, Surrey, SM2 5NG, UK; Falkland Islands Government Health and Social Services, King Edward VII Memorial Hospital, Stanley, Falkland Islands

**Keywords:** cancer incidence, Falkland Islands, cancer screening

## Abstract

Cancer incidence in the Falkland Islands, 1989–2000, was compared with rates in England and Wales, from which most Islanders originate. Colon and rectum cancer incidence was significantly raised 1989–93 but greatly reduced after 1994, when colonoscopic screening in high-risk families and sigmoidoscopic screening in the general population were introduced. http://www.bjcancer.com © 2001 Cancer Research Campaign


*British Journal of Cancer* (2001) **85**(9), 1332–1334
© 2001 Cancer Research Campaign
doi: 10.1054/ bjoc.2001.2099, available online at http://www.idealibrary.com on IDEAL

Short Communication

### Cancer incidence in the Falkland Islands

**AJ Swerdlow[1], B Elsby[2] and Z Qiao[1]**

[1]Section of Epidemiology, D. Block, Institute of Cancer Research, Cotswold Rd, Sutton, Surrey SM2 5NG, UK; and [2]Falkland Islands Government, Health and Social Services, King Edward VII Memorial Hospital, Stanley, Falkland Islands

**Summary** Cancer incidence in the Falkland Islands, 1989–2000, was compared with rates in England and Wales, from which most Islanders originate. Colon and rectum cancer incidence was significantly raised 1989–93 but greatly reduced after 1994, when colonoscopic screening in high-risk families and sigmoidoscopic screening in the general population were introduced. © 2001 Cancer Research Campaign http://www.bjcancer.com

**Keywords**: cancer incidence; Falkland Islands; cancer screening

The Falkland Islands are a self-governing British territory situated in the South Atlantic Ocean, 300 miles from the southern tip of South America. The islands have been under British control since 1833, and the population of 2300 is almost entirely of British (mainly English and Welsh) origin, with about half having some ancestry from the 38 original settlers. The main occupations on the Islands are sheep-farming, government employment and service industries. Cancer incidence rates in these isolated Islands are of interest both in a genetic context and because of the particular exposures and circumstances of the population: they have had a diet high in animal fat and low in fresh fruit and vegetables, and have recently had sudden large influxes of temporary residents.

### MATERIALS AND METHODS

Cases of cancer occurring in the Falkland Islands from 1 January 1989 to 31 December 2000 were ascertained from the records of the only general practice in the Islands, and the knowledge of medical staff who have worked there throughout the period. In addition, all death certificates since January 1989 were searched for mention of cancer, and the incidence dates of these cancers then ascertained to determine whether they were within the study period. All of the cancers diagnosed had been confirmed by histology in a UK laboratory. Demographic information about the population at the Census of 26 April 1996 was obtained from the Office of the Governor of the Islands. The population data included a count of persons who had been resident in the Islands for at least 5 years at the time of the census. We restricted our analyses to cancers in the population who met this requirement, because many military personnel and civilians spend short periods in the Islands, but their cancer incidence is unlikely to relate to factors occurring there, and these cancers would not necessarily be diagnosed within the Islands' medical system. Cancer sites were coded according to the Ninth revision of the International Classification of Diseases (WHO, 1977).

Rates of cancer incidence in the Falklands were calculated using indirect age and sex standardization, with rates in England and Wales in 1993 as the standard. Standardized incidence ratios (SIRs) were calculated to compare Falklands rates with published cancer incidence rates in England and Wales in 1993. England and Wales was selected as the comparison population because the population of the Falklands are largely of English and Welsh origin. Rates in 1993 were chosen because this is the most recent year for which England and Wales cancer registration data are available, and is as near as practical to the midpoint of the period of the Falklands data. We omitted non-melanoma skin cancers from the study because registration of them in England and Wales is substantially incomplete and the same may be true for the Falklands data if any cases were not biopsied.

### RESULTS

Nine hundred and thirty males and 792 females aged 15 years above were present in the Falklands at the census in 1996 and had been resident there for 5 years or more. Eighty-five cancers were incident in this population during January 1989 to December 2000, giving an overall incidence rate of 485 per 100 000 per year – similar to that expected from rates in England and Wales (Table 1). There were significantly raised risks in the Falklands compared with England and Wales for cancers of the small intestine, larynx, and breast in males, and a borderline significantly raised risk of Hodgkin's disease; the risk of lymphomas overall was significantly raised (SIR = 2.21 (95% CI 1.05–4.64); not in table). There were no cases of cancer of the cervix, but this deficit was not significant. One cancer, a Wilms' tumour, occurred in a child (not in table). Although the rate of colorectal cancer was not significantly raised, we examined the time distribution of cases because screening was introduced in the Islands in 1994 (see Discussion): in 1989–93, nine cases occurred, with an SIR of 2.18 (95% CI 1.13–4.18) compared with rates in England and Wales; in 1994 five cases were diagnosed, two directly at screening; and in 1995–2000 there was only one further case (SIR = 0.20 (95% CI 0.03–1.43)).

*Received 22 June 2001*
*Revised 10 August 2001*
*Accepted 13 August 2001*

*Correspondence to:* AJ Swerdlow

**Table 1** Cancer incidence in the Falkland Islands, 1989–2000, in persons resident there for at least 5 years, compared with England and Wales, 1993

| ICD9 code, cancer site | | No. of cases in Falklands | SIR (95% CI)[a] |
|---|---|---|---|
| 150 | Oesophagus | 4 | 1.97 (0.74–5.26) |
| 151 | Stomach | 3 | 0.89 (0.29–2.76) |
| 152 | Small intestine | 2 | 13.34 (3.34–53.34)*** |
| 153–4 | Colon and rectum | 15 | 1.51 (0.91–2.51) |
| 157 | Pancreas | 1 | 0.49 (0.07–3.46) |
| 161 | Larynx | 3 | 3.65 (1.18–11.33)* |
| 162 | Lung | 14 | 1.07 (0.63–1.80) |
| 172 | Melanoma | 3 | 1.59 (0.51–4.91) |
| 174 | Breast, female | 11 | 1.09 (0.60–1.96) |
| 174 | Breast, female, ages < 50 | 3 | 1.04 (0.33–3.21) |
| 175 | Breast, male | 2 | 18.64 (4.66–74.54)*** |
| 179,182 | Corpus uteri and uterus unspecified | 2 | 1.52 (0.38–6.07) |
| 180 | Cervix | 0 | 0 (0–3.02) |
| 183 | Ovary | 2 | 1.19 (0.30–4.74) |
| 183 | Ovary, ages < 50 | 1 | 2.69 (0.38–19.09) |
| 184 | Other female genital | 1 | 3.54 (0.50–25.16) |
| 185 | Prostate | 7 | 1.02 (0.49–2.15) |
| 186 | Testis | 2 | 2.89 (0.72–11.54) |
| 188 | Bladder | 2 | 0.45 (0.11–1.79) |
| 191–2 | Brain and other N.S. | 1 | 0.69 (0.10–4.91) |
| 193 | Thyroid | 1 | 2.76 (0.39–19.60) |
| 200,202 | Non-Hodgkin's lymphoma | 5 | 1.90 (0.79–4.55) |
| 201 | Hodgkin's disease | 2 | 3.60 (0.90–14.41) |
| 204–8 | Leukaemia | 1 | 0.56 (0.08–3.94) |
| 140–172, 174–208 | All cancers except non-melanoma skin cancer | 85 | 1.08 (0.88–1.34) |

$^*P < 0.05$ $^{**}P < 0.01$ $^{***}P < 0.001$.
[a]Falklands compared with England and Wales, 1993, age- and sex-standardized.

### DISCUSSION

The population of the Falkland Islands is of interest in terms of cancer risk for several reasons. Many of the population come from families who have lived in the Islands for several generations, and have common ancestors. The Islands are physically isolated, but there have been large influxes of temporary residents (about 4500 troops during the First and Second World Wars, a construction workforce of 3000–4000 in 1982–5 to construct a runway and airfield complex, and about 1500–2000 troops since the Falklands War of 1982). The diet has been unusually high in animal fat and deficient in fresh fruit and vegetables – before the Falklands War, many individuals received free mutton and ate it three times a day, and until relatively recently the arrival of fresh fruit was an uncommon, newsworthy, event.

Certain of the excesses of malignancy seen in the study seem explicable by one of these factors and others might be so, although it should be noted that numbers of cases are small, and chance may be responsible for some of the findings. There have in the past been large numbers of colonic cancers in three or four families with high risk at a young age, and who since 1994 have received regular screening by colonoscopy (uptake by all but one individual). In addition, the whole Island's population aged 56–75 years was offered extended flexible sigmoidoscopy in 1994 (Saunders et al, 1995), and subsequent colonoscopy if needed, and the same offer was made to other Islanders later as they reached age 56; uptake has been over 90%. It is notable that whereas nine cases (two in high risk families) of colorectal cancer occurred in the 5 years before screening was initiated, there has been only one (not in a high risk family) in the 6 years since, and the two cases found at screening in 1994 would presumably otherwise have been diagnosed during the next six years. Although based on small numbers, the decrease in incidence is encouraging. Future data are needed to confirm whether screening has indeed been effective.

The excess of small intestinal tumours, although based on only two cases, was statistically significant; the cases were not from families at high risk of colorectal cancer. The highly significant excess of male breast cancers (based on two cases) raises the possibility of a genetic basis, as male breast cancer has been found associated with the *BRCA2* gene (Thorlacius et al, 1996; Friedman et al, 1997). The cases, however, were not from families in which breast cancer or ovarian cancer are known to have occurred in women; they were not known to have any ancestors in common, and had not been tested for *BRCA2*. There was no excess of breast cancer in young women or of ovarian tumours in the Islands.

There were no cases of cervical cancer, although the deficit was not significant; 2-yearly screening is offered to women in the Islands aged 18 to 65 years. There was a significantly raised risk of lymphomas overall, based on 7 cases. Epidemiological evidence suggests that Hodgkin's disease is caused by an infection which is more likely to cause the disease if acquired late, rather than in childhood (Mueller, 1996). This raises the possibility that the influx of military and other outsiders during and after the Falklands War might have introduced an infection to individuals who had not previously experienced it. Both cases of Hodgkin's disease occurred several years after the War. Non-Hodgkin's lymphoma has been shown to be associated with various forms of immunodeficiency, and may have a viral component in aetiology. None of the lymphomas were in persons who had been therapeutically immunosuppressed or had HIV infection. Childhood leukaemia incidence in the Islands after the influx of large numbers of construction workers and troops is of interest in rela-

*© 2001 Cancer Research Campaign*

tion to the theory of an infectious origin of this malignancy facilitated by population mixing (Kinlen, 1996). No cases occurred, but the population of children is small and the expected incidence is tiny.

Alcohol and tobacco consumption in the Islands has been high, and the significant excess of laryngeal cancers may reflect this. It is notable that despite the absence of air pollution and industrial occupations in the Islands, lung cancer risks were close to those in England and Wales.

Cancer rates in the Falklands need continued monitoring, especially with regard to cancers of potential genetic origin in this long-established island population, the possible excess of lymphomas, and the effect of screening on rates of colorectal cancer.

### ACKNOWLEDGEMENTS

We thank the Governor of the Falkland Islands for census data, Mrs Sylvia Summers for her invaluable local knowledge and help in carrying out searches, Mrs M Snigorska for secretarial help, Dr I Frayling for advice, and Dr M Gore for putting us in touch with each other.

### REFERENCES

Friedman LS, Gayther SA, Kurosaki T, Gordon D, Noble B, Casey G, Ponder BAJ and Anton-Culver H (1997) Mutational analysis of *BRCA1* and *BRCA2* in a male breast cancer population. *Am J Hum Genet* **60**: 313–319

Kinlen LJ (1996) Epidemiological evidence for an infective basis in childhood leukaemia. *J R Soc Health* **116**: 393–399

Mueller NE (1996) Hodgkin's disease. In: *Cancer epidemiology and prevention* 2nd edn, Schottenfeld D and Fraumeni JF Jr (eds) pp 893–919. Oxford University Press: New York

Saunders BP, Elsby B, Boswell AM, Atkin W and Williams CB (1995) Intravenous antispasmodic and patient-controlled analgesia are of benefit for screening flexible sigmoidoscopy. *Gastrointest Endosc* **42**: 123–127

Thorlacius S, Olafsdottir G, Tryggvadottir L, Neuhausen S, Jonasson JG, Tavtigian SV, Tulinius H, Ögmundsdottir HM and Eyfjörd JE (1996) A single *BRCA2* mutation in male and female breast cancer families from Iceland with varied cancer phenotypes. *Nat Genet* **13**: 117–119

World Health Organization (1977) *Manual of the International Statistical Classification of diseases, injuries, and causes of death*. 9th Revision. WHO: Geneva

*© 2001 Cancer Research Campaign*

